# Simplified predictive scores for thrombosis and bleeding complications in newly diagnosed acute leukemia patients

**DOI:** 10.1186/s12959-023-00506-2

**Published:** 2023-06-08

**Authors:** Weerapat Owattanapanich, Tarinee Rungjirajittranon, Apichaya Jantataeme, Smith Kungwankiattichai, Theera Ruchutrakool

**Affiliations:** 1 Division of Hematology, Department of Medicine, Faculty of Medicine Siriraj Hospital, Mahidol University, 2 Wanglang Road, Bangkoknoi, Bangkok, 10700 Thailand; 2 Center of excellence of Siriraj Adult Acute Myeloid/Lymphoblastic Leukemia (SiAML), Siriraj Hospital, Mahidol University, Bangkok, Thailand

**Keywords:** Acute leukemia, Bleeding, D-dimer, DIC, Disseminated intravascular coagulation, Hemorrhage, Thrombosis

## Abstract

**Background:**

Bleeding and thrombotic complications are the leading causes of death in acute leukemia patients. The Conventional International Society of Thrombosis and Haemostasis Disseminated Intravascular Coagulation (ISTH DIC) scoring system is utilized to assess DIC diagnoses in various conditions. Nevertheless, limited studies have tested the system’s accuracy in predicting thrombo-hemorrhagic events in individuals with acute leukemia. This study aimed to (1) validate the ISTH DIC scoring system and (2) propose a new Siriraj Acute Myeloid/Lymphoblastic Leukemia (SiAML) bleeding and thrombosis scoring system for thrombohemorrhagic risk assessment in acute leukemia.

**Methods:**

We conducted a retro-prospective observational study of newly diagnosed acute leukemia patients between March 2014 and December 2019. We recorded thrombohemorrhagic episodes within 30 days postdiagnosis and DIC profiles, including prothrombin time, platelet level, D-dimer, and fibrinogen. The sensitivities, specificities, positive and negative predictive values, and areas under receiver operating characteristic curves for the ISTH DIC and SiAML scoring systems were calculated.

**Results:**

In all, 261 acute leukemia patients were identified: 64% with acute myeloid leukemia, 27% with acute lymphoblastic leukemia, and 9% with acute promyelocytic leukemia. Overall bleeding and thrombotic events were 16.8% and 6.1%, respectively. With a cutoff of 5 for the ISTH DIC score, the sensitivity and specificity for bleeding prediction were 43.5% and 74.4%, respectively, while the corresponding values for thrombotic prediction were 37.5% and 71.8%, respectively. D-dimer > 5000 µg FEU/L and fibrinogen ≤ 150 mg/dL were significantly associated with bleeding. A SiAML-bleeding score was calculated using these factors, with a sensitivity and specificity of 65.2% and 65.6%, respectively. Conversely, D-dimer > 7000 µg FEU/L, platelet > 40 × 10^9^/L, and white blood cell level > 15 × 10^9^/L were significant variables related to thrombosis. Using these variables, we established a SiAML-thrombosis score with a sensitivity and specificity of 93.8% and 66.1%, respectively.

**Conclusions:**

The proposed SiAML scoring system might be valuable for prognosticating individuals at risk for bleeding and thrombotic complications. Prospective validation studies are needed to verify its usefulness.

**Supplementary Information:**

The online version contains supplementary material available at 10.1186/s12959-023-00506-2.

## Introduction

Acute leukemia patients always require prompt treatment to avert the otherwise severe natural course of the disease. Moreover, swift action helps to stave off its complications: hyperleukocytosis syndrome, tumor lysis syndrome, severe infection, life-threatening bleeding, and thrombosis [1].

One of the major explanations for thrombohemorrhagic complications is disseminated intravascular coagulation (DIC) [1, 2]. The International Society of Thrombosis and Haemostasis (ISTH) subcommittees on DIC and perioperative and critical-care thrombosis and hemostasis jointly declared that there is high-level evidence that DIC is a complication of acute leukemia [3]. In addition, Thai Acute Leukemia Study Group data revealed that approximately 5% of Thai acute myeloid leukemia (AML) patients presented with DIC. The data also showed that the incidence of DIC was more significant among patients with acute promyelocytic leukemia (APL; 37.7%) than among those without APL (1.1%) [2]. Another study reported that 12% of patients with acute lymphoblastic leukemia (ALL) displayed DIC at presentation [4]. Real-world data from a cohort study showed that approximately one-quarter (26%) of AML patients had bleeding manifestations while receiving induction chemotherapy. Significant risk factors were lower platelet numbers and elevated prothrombin time (PT) [5]. Although thrombosis has been considered less common than bleeding in patients with acute leukemia, numerous studies have reported incidences of thrombosis ranging between 2 and 13% [6–12]. Moreover, the thrombotic complications arising from DIC can be arterial or venous [6].

The ISTH DIC scoring system has been widely acknowledged as a reliable screening tool to detect DIC, regardless of the cause [13]. Four parameters are evaluated at diagnosis: platelet level, fibrinogen, D-dimer, and PT [13]. It could be postulated that not all the variables are of equal importance. Thus, thrombocytopenia might be too common a manifestation to be a distinguishing factor. On the other hand, hypofibrinogenemia is an unusual presentation except in APL patients [6]. Only a limited number of studies have evaluated the clinical relevance of bleeding and thrombosis in acute leukemia patients using the ISTH DIC scoring system [14, 15].

We reviewed the literature and identified a need for a DIC prediction tool for patients with acute leukemia. This study therefore had the following 3 aims:

- establish the incidence of overt DIC in acute leukemia subtypes
- validate the standard criteria of the ISTH DIC scoring system for predicting 30-day thrombohemorrhagic events
- propose a simplified predictive scoring system for bleeding and thrombosis complications in these patients

## Materials and methods

### Study design and participants

This was a retro-prospective observational study of newly diagnosed acute leukemia patients. They were treated at Siriraj Hospital, Mahidol University, Bangkok, Thailand, between March 2014 and December 2019. To be eligible for the study, participants had to be 15 or older and have been recently diagnosed with acute leukemia, including ALL, APL, and AML. Excluded were patients (1) with familial thrombophilia, (2) using antiplatelet or anticoagulant agents before their acute leukemia diagnosis, or (3) without DIC-related laboratory results at diagnosis. We collected baseline patient characteristics, thrombohemorrhagic episodes occurring within 30 days postdiagnosis, and initial investigations (complete blood count, PT, activated partial thromboplastin time, fibrinogen, and D-dimer at diagnosis and/or before starting chemotherapy). The Thai Clinical Trial Registry number was TCTR20230404004.

### Definition of outcomes

The ISTH DIC score, based on the ISTH scoring system for DIC [13], was determined for all eligible patients at diagnosis. A DIC score of ≥ 5 was defined as overt DIC [13].

The 30-day (early) thrombohemorrhagic episodes after acute leukemia diagnosis were recorded. This period was chosen because it corresponded with the duration of admission for induction chemotherapy. Acute thrombotic events included acute arterial and venous thrombosis. The diagnoses of thrombosis had to be confirmed by imaging studies, such as compression Doppler ultrasonography, computed tomography pulmonary angiogram, or computed tomography. The definition of major bleeding specified in the DIC criteria of the ISTH was used. The term encompasses fatal bleeding, symptomatic bleeding at a vital area or organ, and bleeding causing a drop in hemoglobin level of ≥ 2 g/dL or necessitating a transfusion of two or more units of whole blood or red cells [16].

### Statistical analysis

Continuous data with a normal distribution are presented as the means and standard deviations. The continuous data were compared using unpaired Student’s t-tests or Mann–Whitney U tests. Categorical data are summarized as frequencies and percentages. Categorical data were analyzed with between-group comparisons using the chi-squared or Fisher’s exact test. The univariate and multivariate predictors of thrombohemorrhagic events were evaluated using logistic regression analysis (backward stepwise method), with results expressed as odds ratios (ORs) and 95% confidence intervals (CIs). The sensitivities, specificities, positive predictive value (PPV) and negative predictive value (NPV), and areas under receiver operating characteristic curves (AUC) for the optimal cutoff ISTH DIC score and the proposed bleeding and thrombosis prediction scores were calculated. The proposed scores were internally validated with the bootstrap resampling technique (1000 replicates). A 2-sided probability (*P*) < 0.05 was considered statistically significant. PASW Statistics for Windows, version 18.0 (SPSS Inc, Chicago, IL, USA) was utilized for the data analyses.

## Results

### Baseline characteristics

In all, 261 acute leukemia patients were enrolled. Their median age was 49 years (interquartile range, 35–61 years), with a slight male predominance (52.1%). The most common acute leukemia subtype was AML (64.0%), followed by ALL (26.8%) and APL (9.2%). Forty-four patients had early major bleeding complications. The common incidents were intracerebral hemorrhage (8 patients), retinal hemorrhage (8 patients), upper gastrointestinal bleeding (7 patients), and lower gastrointestinal bleeding (6 patients). Sixteen patients had early thrombotic complications. They were acute cerebral infarction (7 patients), acute pulmonary embolism (5 patients), deep vein thrombosis (2 patients), splenic infarction (2 patients), and acute arterial occlusion (1 patient; Table 1).

**Table 1:** Baseline characteristics of patients with acute leukemia

Factor	Total (*N* = 261) (100%)	ALL (A) (*N* = 70) (26.8%)	APL (B) (*N* = 24) (9.2%)	AML (C) (*N* = 167) (64.0%)	*P*	*P* for multiple comparisons		
						A vs. B	A vs. C	B vs. C
**Sex**					0.314	0.151	0.280	0.398
**-**Female	125 (47.9%)	29 (41.4%)	14 (58.3%)	82 (49.1%)				
-Male	136 (52.1%)	41 (58.6%)	10 (41.7%)	85 (50.9%)				
**Median age (IQR) (years)**	49 (35–61)	35 (22–58)	43 (34–48)	55 (40–63)	** < 0.001**	0.336	** < 0.001**	**0.001**
**Clinical features of major bleeding (WHO grade 4)**								
-Oropharyngeal	4 (1.5%)	4 (5.7%)	0 (0%)	0 (0%)				
-Epistaxis	3 (1.1%)	2 (2.9%)	0 (0%)	1 (0.6%)				
-Hemoptysis	5 (1.9%)	1 (1.4%)	1 (4.2%)	3 (1.8%)				
-UGIB	7 (2.7%)	2 (2.9%)	0 (0%)	5 (3.0%)				
-LGIB	6 (2.3%)	3 (4.3%)	0 (0%)	3 (1.8%)				
-Hematuria	4 (1.5%)	3 (4.3%)	1 (4.2%)	0 (0%)				
-Vaginal bleeding	4 (1.5%)	1 (1.4%)	2 (8.2%)	1 (0.6%)				
-Muscle hematoma	1 (0.4%)	0 (0%)	1 (4.2%)	0 (0%)				
-Retinal hemorrhage	8 (3.1%)	5 (7.1%)	0 (0%)	3 (1.8%)				
-Intracerebral hemorrhage	8 (3.1%)	1 (1.4%)	2 (8.3%)	5 (3.0%)				
-Overall bleeding events (numbers of patients)	50 (44 patients)	22 (18 patients)	7 (6 patients)	21 (20 patients)	**0.019**	0.945	**0.009**	0.107
**Clinical features of thrombosis**								
-Deep vein thrombosis	2 (0.8%)	0 (0%)	0 (0%)	2 (1.2%)				
-Pulmonary embolism	5 (1.9%)	1 (1.4%)	2 (8.3%)	2 (1.2%)				
-Acute arterial occlusion	1 (0.4%)	0 (0%)	1(4.2%)	0 (0%)				
-Splenic infarction	2 (0.8%)	0 (0%)	0 (0%)	2 (1.2%)				
-Acute cerebral infarction	7 (2.7%)	2 (2.9%)	0 (0%)	5 (3.0%)				
-Overall thrombotic events (numbers of patients)	17 (16 patients)	3 (3 patients)	3 (2 patients)	11 (11 patients)	0.719	0.599	0.763	0.670
**Laboratory at diagnosis**								
**Mean ± SD**								
-Hemoglobin (g/L)	78 ± 23	87 ± 24	84 ± 23	74 ± 21	** < 0.001**	0.582	** < 0.001**	**0.035**
**Median (IQR)**								
-WBC (× 10^9^/L)	18.50 (3.96–95.50)	26.72 (4.30–116.63)	4.66 (1.20–26.09)	18.27 (4.52–86.31)	**0.012**	**0.008**	0.476	**0.005**
-Platelet (× 10^9^/L)	40.00 (18.00–81.00)	50.50 (21.00–94.00)	33.00 (20.00–49.00)	37.00 (16.00–73.00)	0.297	0.151	0.210	0.554
-PT (seconds)	14.0 (13.0–15.6)	13.6 (12.5–15.3)	14.1 (13.4–16.8)	14.0 (13.1–15.6)	0.189	0.130	0.127	0.467
-APTT (seconds)	26.1 (23.8–29.4)	26.9 (23.9–28.9)	25.5 (23.8–30.2)	25.8 (23.5–29.5)	0.928	0.745	0.725	0.976
-Fibrinogen (mg/dL)	349 (247–453)	338 (219–436)	153 (123–287)	372 (272–484)	** < 0.001**	** < 0.001**	**0.044**	** < 0.001**
-D-dimer (µg FEU/L)	3,259.1 (1,120.0–8,492.5)	4,034.7 (1,338.6–9,145.9)	9,308.5 (5,490.2–10,000.0)	2,394.4 (967.5–6,502.0)	** < 0.001**	**0.001**	0.057	** < 0.001**
Median ISTH-DIC scores	3 (2–5)	3 (1–5)	5 (4–6)	3 (2–4)	** < 0.001**	**0.001**	0.479	** < 0.001**
Numbers of overt DIC	75 (28.7%)	20 (28.6%)	15 (62.5%)	40 (24.0%)	** < 0.001**	**0.003**	0.456	** < 0.001**

*Abbreviations*: *ALL* Acute lymphoblastic leukemia, *AML* Acute myeloid leukemia, *APL* Acute promyelocytic leukemia, *APTT* Activated partial thromboplastin time, *DIC* Disseminated intravascular coagulation, *FEU* Fibrinogen equivalent units, *IQR* Interquartile range, *ISTH* The International Society on Thrombosis and Haemostasis, *LGIB* lower gastrointestinal bleeding, *PT* Prothrombin time, *SD* Standard deviation, *UGIB* Upper gastrointestinal bleeding, *WBC* White blood cell

Regarding clinical biochemistry (Table 1), the initial complete blood count revealed a mean hemoglobin level of 78 ± 23 g/L, a median white blood cell (WBC) count of 18.5 × 10^9^/L (3.96–95.5 × 10^9^/L), and a median platelet count of 40.0 × 10^9^/L (18.0–81.0 × 10^9^/L). The coagulation test results showed a median PT of 14.0 s (13.0–15.6 s), median activated partial thromboplastin time of 26.1 s (23.8–29.4 s), median fibrinogen of 349 mg/dL (247–453 mg/dL), and median D-dimer of 3259.1 µg FEU/L (1120.0–8492.5 µg FEU/L). The median ISTH DIC score was 3 (2–5). Approximately a quarter (28.7%) of the patients had overt DIC.

### Comparison of baseline characteristics and outcomes of individual acute leukemia subtypes

In the case of the individual acute leukemia subtype, AML patients were significantly older than ALL and APL patients (*P* < 0.001 and 0.001, respectively). The overall bleeding events occurred most frequently with ALL (25.7%), followed by APL (25.0%) and AML (11.9%). There were no significant differences in the overall thrombotic events of the subtypes (*P* = 0.719). In contrast, APL patients had a significantly higher incidence of overt DIC, calculated by the ISTH DIC scoring system (62.5%), than the ALL and AML patients (*P* = 0.003 and < 0.001, respectively; Table 1).

The median fibrinogen and D-dimer levels significantly differed among acute leukemia subtypes. The median fibrinogen level was lowest for the APL patients, whereas the median D-dimer level was highest in these patients (Table 1).

### Comparison of baseline characteristics of thrombohemorrhagic and non-thrombohemorrhagic groups

A group comparison revealed that the ALL and APL subtypes had higher proportions of patients in the bleeding group than in the nonbleeding group (*P* = 0.033). Moreover, patients with bleeding complications had significantly lower fibrinogen levels (275 vs 363 mg/dL; *P* = 0.004). The median ISTH DIC score of the bleeding group was 4, whereas the score of the nonbleeding group was 3 (*P* = 0.037). More than 40% of the patients in the bleeding group had overt DIC. This value was significantly higher than the proportion of patients without bleeding symptoms who had overt DIC (only 25.6%; *P* = 0.015; Supplementary Data 1).

A comparison of the thrombotic and nonthrombotic cohorts did not reveal any significant discrepancies in the proportions of patients with each acute leukemia subtype, median ISTH DIC scores, or incidence of overt DIC. Nevertheless, the WBC, platelet, and D-dimer values were significantly higher for the patients with thrombotic events (*P* = 0.012, 0.017, and 0.010, respectively; Supplementary Data 2).

### Comparison of ISTH DIC and proposed new scores (SiAML) to predict bleeding and thrombosis in acute leukemia patients

**Fig. 1:**
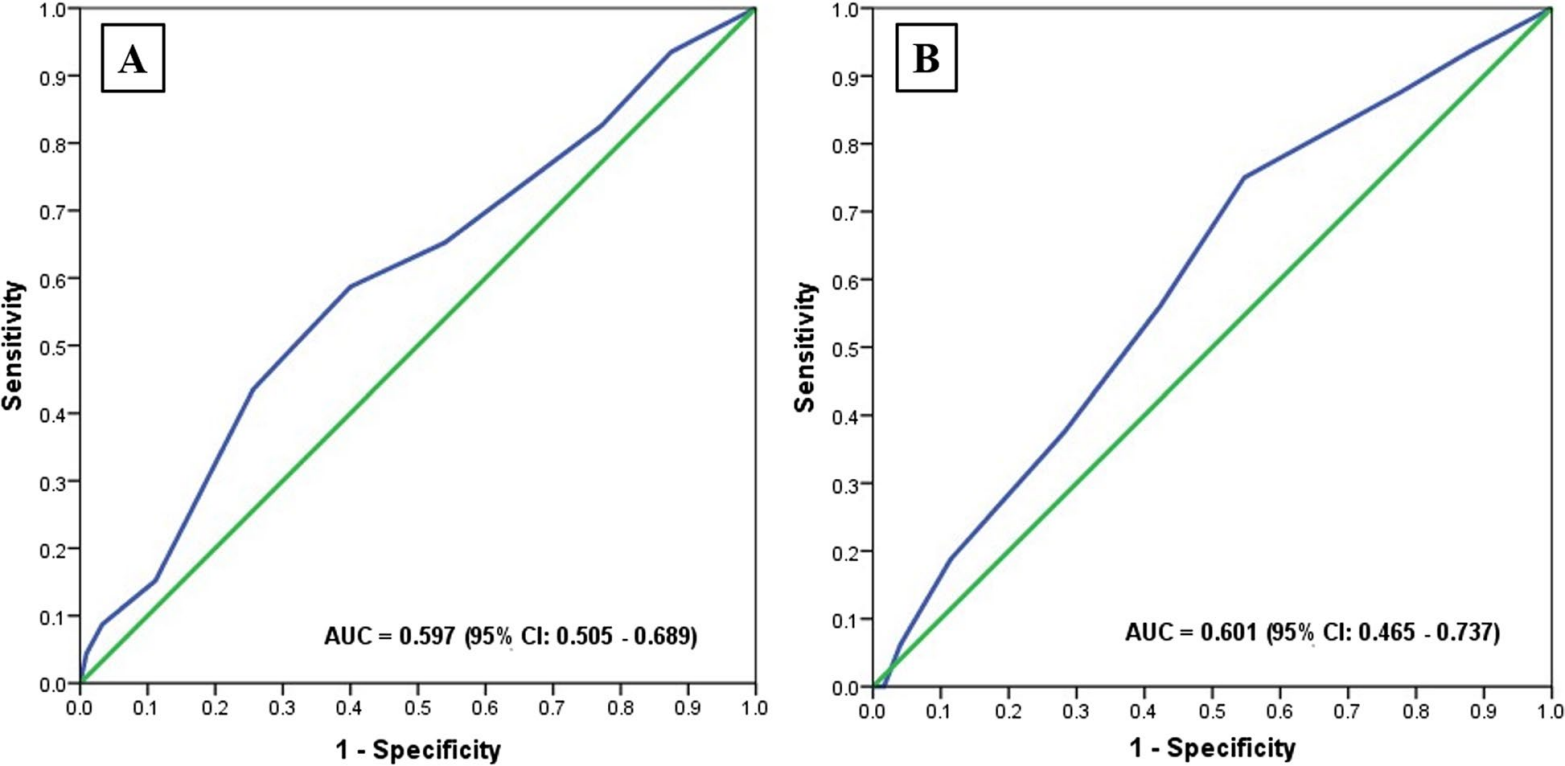
Receiver operating characteristic curves of the ISTH DIC score for predicting (**A**) bleeding and (**B**) thrombosis in patients with acute leukemia

A receiver operating characteristic curve analysis tested the power of the bleeding prediction of the ISTH DIC scoring system using the conventional cutoff (≥ 5). The AUC for bleeding prediction was 0.59 (95% confidence interval [CI], 0.51–0.69; Fig. 1A). The sensitivity and specificity of the score were 43.5% and 74.4%, respectively (Table 2). These data enabled us to calculate a new cutoff for detecting bleeding complications. The most appropriate cutoff was 4 points, with a sensitivity and specificity of 58.7% and 60.0%, respectively (Table 2). Concerning the AUC for thrombotic prediction for overt DIC, the level was 0.60 (95% CI, 0.47–0.74; Fig. 1B)**.** The sensitivity and specificity of the cutoff were 37.5% and 71.8%, respectively (Table 2). As with the bleeding prediction cutoff, 4 points was the most suitable level for predicting thrombotic events, with a sensitivity and specificity of 56.3% and 57.6%, respectively (Table 2).

**Table 2:** Sensitivity, specificity, positive predictive value, negative predictive value, and accuracy of the ISTH-DIC score to predict bleeding and thrombotic complications

Logistic model	Cutoff	Number of overall events (%)	Bleeding or thrombotic events N (%)	Sensitivity (95% CI)	Specificity (95% CI)	PPV (95% CI)	NPV (95% CI)	Accuracy (95% CI)
ISTH-DIC score	≥ 4	86 (40.0)	27 (58.7)	58.7%	60.0%	23.9%	87.2%	59.8%
(bleeding)	< 4	129 (60.0)	19 (41.3)	(43.2–73.0)	(53.1–66.6)	(16.4–32.8)	(80.7–92.1)	(53.8–65.7)
ISTH-DIC score (bleeding)	≥ 5	55 (25.6)	20 (43.5)	43.5%	74.4%	26.7%	86.0%	68.9
	< 5	160 (74.4)	26 (56.5)	(28.9–58.9)	(68.0–80.1)	(17.1–38.1)	(80.2–90.7)	(63.4–74.58)
ISTH-DIC score	≥ 4	104 (42.4)	9 (56.2)	56.3%	57.6%	8.0%	95.3%	57.5%
(thrombosis)	< 4	141 (57.6)	7 (43.8)	(29.9–80.2)	(51.1–63.8)	(3.7–14.6)	(90.5–98.1)	(51.5–63.5)
ISTH-DIC score	≥ 5	69 (28.2)	6 (37.5)	37.5%	71.8%	8.0%	94.6%	69.7%
(thrombosis)	< 5	176 (71.8)	10 (62.5)	(15.2–64.6)	(65.8–77.4)	(3.0–16.6)	(90.3–97.4)	(64.2–75.3)

*Abbreviations*: *NPV* Negative predictive value, *PPV* Positive predictive value

**Fig. 2:**
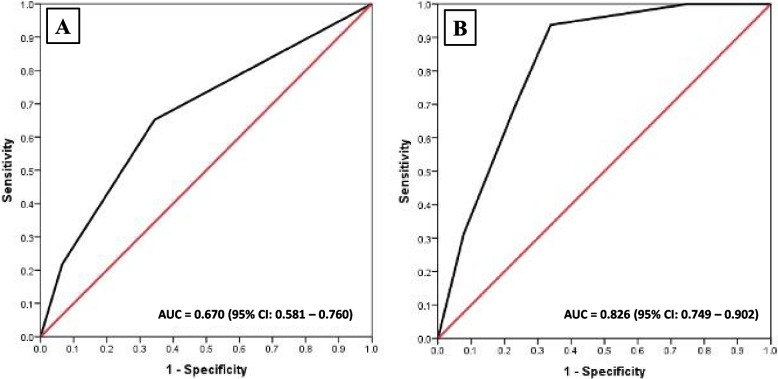
Receiver operating characteristic curves of the proposed SiAML score for predicting (**A**) bleeding and (**B**) thrombosis in patients with acute leukemia

**Fig. 3:**
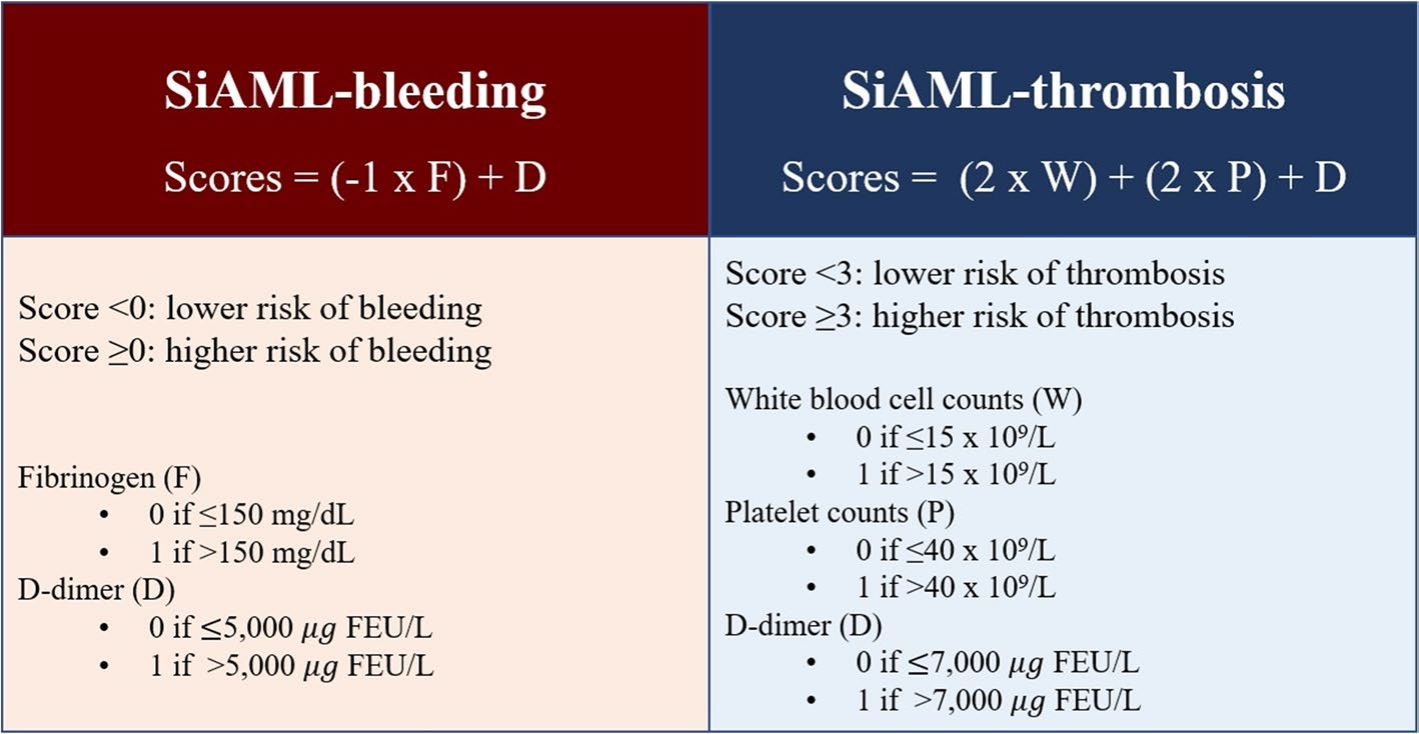
SiAML bleeding and thrombotic scores

Given the limitations of the ISTH DIC scoring system stated above, we decided to design a simplified scoring system that employed only the significant variables influencing bleeding and thrombosis complications. To this end, univariate and multivariate analyses were used to evaluate the possible factors related to bleeding complications. After evaluating the optimal cutoff value to distinguish between the bleeding and non-bleeding groups, it was found that a D-dimer level > 5000 µg FEU/L and a fibrinogen level ≤ 150 mg/dL were significantly associated with hemorrhage. Their ORs were 2.17 (95% CI, 1.06–4.44) and 0.29 (95% CI, 0.12–0.75), respectively (Table 3). A new SiAML-bleeding score was developed based on only these 2 biologically significant factors. The AUC for bleeding prediction was 0.67 (95% CI, 0.58–0.76; Fig. 2A). A cutoff of 0 gave an accuracy of 65.5%, sensitivity of 65.2%, specificity of 65.6%, PPV of 28.8%, and NPV of 89.9%. The proposed SiAML-bleeding score is illustrated in Fig. 3.

**Table 3:** Factors associated with bleeding complications

Factors	Univariate analysis		Multivariate analysis OR (95% CI) *P*
	OR (95% CI)	*P*	
Age (> 60 vs ≤ 60 years)	1.05 (0.51–2.18)	0.891	N/A
APL vs non-APL subtype	1.24 (0.44–3.51)	0.687	N/A
D-dimer (> 5000 vs ≤ 5000 µg FEU/L)	2.96 (1.54–5.71)	**0.001**	2.17 (1.06–4.44) ***P*** **= 0.035**
Platelet level (> 40 vs ≤ 40 × 10^9^/L)	0.82 (0.43–1.55)	0.535	N/A
WBC level (≤ 15 vs > 15 × 10^9^/L)	1.13 (0.60–2.13)	0.709	N/A
Fibrinogen (> 150 vs ≤ 150 mg/dL)	0.20 (0.08–0.46)	** < 0.001**	0.29 (0.12–0.75) ***P*** **= 0.010**

Remark: multivariate analyses were performed with a logistic regression model

*Abbreviations*: *CI* Confidence interval, *N/A* Not available, *OR* Odds ratio, *WBC* White blood cell

Regarding factors correlated with thrombosis, a D-dimer level > 7000 µg FEU/L, platelet level > 40 × 10^9^/L, and WBC level > 15 × 10^9^/L were significant factors by multivariate analysis. The corresponding ORs were 3.49 (95% CI, 1.16–10.44), 5.88 (95% CI, 1.57–21.98), and 5.93 (95% CI, 1.27–27.59), respectively (Table 4). The proposed SiAML-thrombosis score was based on these significant factors, and the most appropriate cutoff was 3 (Fig. 3). The AUC for thrombosis prediction was 0.83 (95% CI, 0.75–0.90; Fig. 2B). The accuracy was 67.8%. The sensitivity and specificity were 93.8% and 66.1%, respectively, while the PPV and NPV were 15.3% and 99.4%, respectively. When comparing the ISTH DIC score to the proposed SiAML scores, it was found that the SiAML scores exhibited higher sensitivity, specificity, and AUC. Moreover, the newly proposed scores were easier to apply to acute leukemia patients due to the utilization of only a few significant factors associated with the disease.

**Table 4:** Factors associated with thrombotic complications

Factors	Univariate analysis		Multivariate analysis OR (95% CI) *P*
	OR (95% CI)	*P*	
Age (> 60 vs ≤ 60 years)	0.98 (0.31–3.16)	0.978	N/A
APL vs non-APL subtype	1.43 (0.31–6.70)	0.651	N/A
D-dimer (> 7,000 vs ≤ 7,000 μg FEU/L)	3.28 (1.18–9.15)	**0.023**	3.49 (1.16–10.44) ***P*** **= 0.026**
Platelet level (> 40 vs ≤ 40 × 10^9^/L)	4.74 (1.32–17.05)	**0.017**	5.88 (1.57–21.98) ***P*** **= 0.008**
WBC level (> 15 vs ≤ 15 × 10^9^/L)	6.94 (1.55–31.20)	**0.011**	5.93 (1.27–27.59) ***P*** **= 0.023**
Fibrinogen (> 100 vs ≤ 100 mg/dL)	0.19 (0.04–1.06)	0.059	N/A

Remark: multivariate analyses were performed with a logistic regression model

*Abbreviations*: *APL* Acute promyelocytic leukemia, *CI* Confidence interval, *N/A* Not available, *OR* Odds ratio, *WBC* White blood cell

Using the bootstrap method, we internally validated our score. The estimated AUC for predicting probabilities of bleeding and thrombotic outcomes were 0.520 (95% CI, 0.403–0.636) and 0.758 (95% CI, 0.669–0.847), respectively. The data indicate that the SiAML-bleeding and SiAML-thrombosis scores can detect bleeding and thrombosis complications in acute leukemia patients.

## Discussion

Acute leukemia patients frequently present with thrombohemorrhagic complications. Patients who developed DIC at diagnosis were at risk for thrombohemorrhagic events and death [17]. The reported incidence of DIC, calculated by the ISTH DIC criteria, has varied between 8 and 90%, depending on the leukemia subtype [6, 18–20]. Numerous studies have demonstrated that APL patients have the highest rates of DIC, with up to 90% of cases meeting the DIC criteria [18–20]. Similar to our study outcomes, more than half of APL (62.5%) patients had DIC at diagnosis. Since consumptive coagulopathy is the primary mechanism of DIC pathogenesis, it is not surprising that our APL patients had the lowest fibrinogen level and the highest D-dimer level of the subtypes [21]. However, our data showed that the highest frequencies of bleeding symptoms were found in both APL and ALL patients. An earlier study demonstrated that ALL patients with a fibrinogen level < 100 mg/dL had a higher risk of bleeding complications [22]. In the case of the patients in our cohort, hypofibrinogenemia was also a significant factor in the bleeding group. Moreover, patients with bleeding symptoms had a higher ISTH DIC score and more patients with overt DIC.

Thrombosis has been observed to have a much lower incidence than bleeding complications, with a rate of less than 10% in most studies [6, 23, 24]. The risk of thrombosis has been reported to be higher in ALL patients receiving L-asparaginase at diagnosis [24, 25]. Our study’s incidence rate was approximately 6%, without any significant difference between the acute leukemia subtypes. Interestingly, the median ISTH DIC scores and the number of patients with overt DIC were not significantly different between patients with and without thrombotic events. Nonetheless, an increased D-dimer, WBC, and platelet count significantly influenced thrombotic complications in our study. Libourel et al. demonstrated that the relationship between DIC and thrombosis with elevated D-dimer was the leading factor in both test and validation cohorts [6]. In their study, the WBC count was also higher in patients with DIC [6]. Furthermore, a higher platelet level was significantly associated with an increased risk of early venous thromboembolism development in an AML study [23]. A recent study also showed that an elevated D-dimer level was a significant variable for ALL patients [26]. More specifically, patients with a D-dimer level ≥ 4 gμ/mL at diagnosis had an increased risk of venous and arterial thrombosis during the first 100 days [26]. Another study showed that weekly D-dimer monitoring helped detect APL patients with silent thrombosis [12].

Several DIC conditions can be assessed using the ISTH DIC scoring system [3]. However, at this writing, only limited studies have validated the clinical relevance between ISTH DIC scores and thrombohemorrhagic complications. Unexpectedly, our study found that the traditional cutoff for overt DIC (≥ 5) failed to demonstrate overall test accuracy for bleeding and thrombosis predictions. Consequently, a new cutoff (≥ 4) was used, resulting in the score’s sensitivity in predicting bleeding and thrombosis improving slightly. Paterno et al. conducted a study investigating the power of mortality prediction of the ISTH DIC score in AML patients [27]. Their investigation showed that ISTH DIC scores ≥ 4 were associated with 30-day mortality, with 36% of patients dying from thrombohemorrhagic complications [27]. However, in one study, an ISTH DIC score cutoff of ≥ 6 was a new predictor of hemorrhagic death in pediatric APL patients [19]. The situation is that there is no consensus on an appropriate ISTH DIC cutoff score for predicting thrombohemorrhagic events in acute leukemia patients. Furthermore, it is not yet certain whether the 4 factors of the ISTH DIC score are truly representative and sufficient for predicting bleeding and thrombosis symptoms.

To resolve these limitations, we created two simplified scores using significant individual factors by separating bleeding from thrombotic outcomes. With the use of only fibrinogen and D-dimer levels for the SiAML-bleeding score, more patients could be detected and ruled out early. This is because of the higher sensitivity and NPV of the SiAML-bleeding score than the ISTH DIC scoring system.

While hypofibrinogenemia from consumptive coagulopathy is the hallmark of bleeding symptoms in acute leukemia patients, high WBC and platelet counts play major roles in thrombosis [28]. Tissue factor and procoagulant molecules from leukemic cells activate platelets and the coagulation system to an active state [24]. Likewise, neutrophil extracellular traps also trigger neutrophils, platelets, and the contact pathway system, resulting in a hypercoagulable state [29]. Consistent with the mechanism of thrombosis, our study demonstrated that patients with higher WBC, platelet, and D-dimer levels were prone to thrombotic complications. Additionally, a previous study conducted on newly diagnosed ALL patients demonstrated that the initial D-dimer level was high, with a median D-dimer value of 2.1 µg/mL [26]. Consistent with our study findings, the baseline D-dimer level in our cohort was 3,259.1 µg FEU/L (equivalent to 1.63 µg/mL). Consequently, the D-dimer cutoff for distinguishing thrombohemorrhagic events should be set higher.The overall test accuracy improved using only these 3 factors, and NPV was nearly 100%. Hence, our data showed that the SiAML-bleeding and SiAML-thrombosis scoring systems might be optional tools for early detection of thrombohemorrhagic complications and prevention of early death in acute leukemia patients.

Some limitations were found in the study. First, there was a small number of patients, especially those with APL. This meant that analyses of the acute leukemia subtypes could not be performed. Moreover, the small patient number might explain the lower incidence of thrombohemorrhagic events than other studies. Second, some patient data and information were missing because the study was retrospective. Finally, external validation studies on different populations and periods are needed to ensure the accuracy and validity of these scores. Ongoing research is underway at our center.

## Conclusions

Early detection of bleeding and thrombosis complications is central to enhancing treatment in newly diagnosed acute leukemia patients. The SiAML scoring system might be a helpful tool for evaluating these patients. However, prospective validation studies are needed before its widespread use in clinics.

## Supplementary Information

**Additional file 1: Supplementary Data 1.** Comparison of the bleeding and non-bleeding groups.

**Additional file 2: Supplementary Data 2.** Comparison of the thrombotic and non-thrombotic groups.

## Data Availability

The datasets used or analyzed during the current study are available from the corresponding author upon reasonable request.
