# Supplementary material for: Simplified predictive scores for thrombosis and bleeding complications in newly diagnosed acute leukemia patients
Source: Thromb J. 2023 Jun 8;21:65. doi: 10.1186/s12959-023-00506-2 (PMC10251548; doi:10.1186/s12959-023-00506-2)
Supplement: Supplementary file 1 — Additional file 1: Supplementary Data 1. Comparison of the bleeding and non-bleeding groups. [file 12959_2023_506_MOESM1_ESM.docx]

**Supplementary Data 1.** Comparison of the bleeding and non-bleeding groups

| **Factor** | **Bleeding group**  **(N = 46) (17.6%)** | **Non-bleeding group**  **(N = 215) (82.4%)** | ***P*** |
| --- | --- | --- | --- |
|  |  |  |  |
| **Sex**  **-**Female  -Male | 19 (41.3%)  27 (58.7%) | 106 (49.3%)  109 (50.7%) | 0.324 |
| **Acute leukemia subtype**  -ALL  -APL  -AML | 19 (41.3%)  5 (10.9%)  22 (47.8%) | 51 (23.7%)  19 (8.8%)  145 (67.4%) | **0.033** |
| **Median age (IQR) (years)** | 47 (35-62) | 50 (35-61) | 0.767 |
| **Mean ± SD**  -Hemoglobin (g/L)  **Median ± IQR**  -WBC (x10^9^/L)  -Platelet (x10^9^/L)  -PT (seconds)  -APTT (seconds)  -Fibrinogen (mg/dL)  -D-dimer (µg FEU/L) | 77.1 ± 20.8  18.93  (4.42-98.14)  32.00  (12.00-71.00)  14.2  (12.9-15.3)  27.2  (23.9-28.9)  275  (149-413)  6,497.03  (1051.0-10 000.0) | 78.6 ± 23.3  18.50  (3.80-93.76)  42.00  (19.00-86.00)  14.0  (13.0-15.6)  25.8  (23.7-29.6)  363  (259-456)  2,868.72  (1125.0-7635.2) | 0.689  0.488  0.137  0.664  0.486  **0.004**  0.081 |
| Median ISTH-DIC scores | 4 (2-5) | 3 (2-5) | **0.037** |
| Numbers of overt DIC | 20 (43.5 %) | 55 (25.6%) | **0.015** |

**Abbreviations**: ALL, acute lymphoblastic leukemia; AML, acute myeloid leukemia; APL, acute promyelocytic leukemia; APTT, activated partial thromboplastin time; DIC, disseminated intravascular coagulation; FEU, fibrinogen equivalent units; IQR, interquartile range; ISTH, the International Society on Thrombosis and Haemostasis; LGIB, lower gastrointestinal bleeding; PT, prothrombin time; SD, standard deviation; UGIB, upper gastrointestinal bleeding; WBC, white blood cell
