# Supplementary material for: Simplified predictive scores for thrombosis and bleeding complications in newly diagnosed acute leukemia patients
Source: Thromb J. 2023 Jun 8;21:65. doi: 10.1186/s12959-023-00506-2 (PMC10251548; doi:10.1186/s12959-023-00506-2)
Supplement: Supplementary file 2 — Additional file 2: Supplementary Data 2. Comparison of the thrombotic and non-thrombotic groups. [file 12959_2023_506_MOESM2_ESM.docx]

**Supplementary Data 2.** Comparison of the thrombotic and non-thrombotic groups

| **Factor** | **Thrombotic group**  **(N = 16) (6.1%)** | **Non-thrombotic group**  **(N = 245) (93.9%)** | ***P*** |
| --- | --- | --- | --- |
|  |  |  |  |
| **Sex**  **-**Female  -Male | 8 (50.0%)  8 (50.0%) | 117 (47.8%)  128 (52.8%) | 0.862 |
| **Acute leukemia subtype**  -ALL  -APL  -AML | 3 (18.8%)  2 (12.5%)  11 (68.8%) | 67 (27.3%)  22 (9.0%)  156 (63.7%) | 0.719 |
| **Median age (IQR) (years)** | 48 (33.5-60.5) | 50 (35-61) | 0.804 |
| **Mean ± SD**  -Hemoglobin (g/L)  **Median ± IQR**  -WBC (x10^9^/L)  -Platelet (x10^9^/L)  -PT (seconds)  -APTT (seconds)  -Fibrinogen (g/L)  -D-dimer (µg FEU/L) | 83.4 ± 32.5  82.06  (23.32-217.25)  70.00  (46.50-138.00)  14.3  (13.8-17.2)  28.4  (25.4-31.7)  3.28  (2.53-4.67)  9,477.50  (3136.06-10 000.00) | 78.0±22.1  15.30  (3.87-90.8)  37.00  (16.00-79.00)  14.0  (12.9-15.5)  25.8  (23.7-29.3)  3.49  (2.46-4.48)  2945.00  (1111.13-7839.54) | 0.523  **0.012**  **0.017**  0.076  0.060  0.981  **0.010** |
| Median ISTH-DIC scores | 4 (2.5-5) | 3 (2-5) | 0.171 |
| Numbers of overt DIC | 6 (37.5%) | 69 (28.2%) | 0.424 |

**Abbreviations**: ALL, acute lymphoblastic leukemia; AML, acute myeloid leukemia; APL, acute promyelocytic leukemia; APTT, activated partial thromboplastin time; DIC, disseminated intravascular coagulation; FEU, fibrinogen equivalent units; IQR, interquartile range; ISTH, the International Society on Thrombosis and Haemostasis; LGIB, lower gastrointestinal bleeding; PT, prothrombin time; SD, standard deviation; UGIB, upper gastrointestinal bleeding; WBC, white blood cell
